# Author Correction: The passband integration properties of Birefringent filter

**DOI:** 10.1038/s41598-021-01719-z

**Published:** 2021-11-09

**Authors:** Xiaofan Wang, Mikhail Leonidovich Demidov, Yuanyong Deng, Haiying Zhang

**Affiliations:** 1 Key Laboratory of Solar Activity, National Astronomical Observatories, Chinese Academy of Sciences, Beijing, 100101 China; 2 Institute of Solar-Terrestrial Physics of Siberian Branch, Russian Academy of Sciences, Irkutsk, 664033 Russia; 3 Nanjing Institute of Astronomical Optics & Technology, National Astronomical Observatories, Chinese Academy of Sciences, Nanjing, 210042 China

Correction to: *Scientific Reports* https://doi.org/10.1038/s41598-021-96126-9, published online 23 August 2021

The original version of this Article contained errors.

In the Abstract,

“This article will give an answer to the question that had been proposed by the previous reachers.”

now reads:

“This article will give a further discussion to the question that had been proposed by the previous researchers.”

In the Introduction, Mathematical Generality of Passband Integration section, and the Summary and Discussion section, Reference Hu, Y. F. & Ai, G. X. (1984) was incorrectly referred to in the singular “Ai”, “he”, or “his”. This has been corrected to “Hu and Ai”, “they”, “their”, or “them”.

In the Introduction, under the subheading ‘Photon constant problem in BF adjustment’,

“There were several formula mistakes in this Chinese publication^18^, but the basic meaning and idea were correct. Ai did not prove the mathematic generality, and also did not pointed out its possible physical meaning, but he numerically verified this “constant” up to 29 elements’ BF by using Chinese computer TQ-16 (120k calculations per second). Of course, it is impossible to build 29 elements’ BF in reality.”

now reads:

“There were some ambiguities in this Chinese publication^18^, especially about the definition of the integration range^18^. But those tiny problems are not important at all when they are compared to the significance of Hu and Ai’s pioneering work which provided a framework for subsequent researches. They used Chinese computer TQ-16 (120k calculations per second) to numerically verify this “constant” up to 29 elements’ BF, even though it is not yet feasible to build in practicality.”

In the Mathematical generality of passband integration section, under the subheading ‘Generality Proof’,

“Hence, the integration invariant properties is still valid at different BF states. His original publication in Chinese have some ambiguity and mistakes about the definition of this integration range^18^. The period corresponding to the thickest BF crystal element absolutely can not be chosen as the integration range (see Fig. 7). This could be the reason why this conservation property was not really proved until this paper. Here we clarify that the integration range should be *the largest period* decided by *the thinnest BF crystal element*!”

now reads:

“Therefore, in the framework of Hu and Ai, our discussion on the BF’s passband integral problem strictly involves three aspects: manufacture, adjustment, and the light source. In the previous observation and data section, both the fixed integral interval and the variable integral interval are calculated and analyzed. In this section, the fixed integration period is applied. Here we clarify that our integration range is **the largest period** decided by the **thinnest BF crystal element**. We will apply the orthogonality of integrated functions and a transformation to discuss the generality of this passband integration, combining the two parts of crystal thickness and phase angle.

The rigorous and concise mathematical demonstration of the conservation of integrated transmission of the tunable Lyot Birefringent Filter can also be realized by expanding the integral into series results and then applying the mathematical induction, which had already been given by Su Dingqiang in 1986^22,23^."

In addition, under the subheading ‘Numerical experiment’,

“The purpose of this transformation is to change the fractional periods into the integer periods, and to simplify the trigonometric function series. The previous subsection could also be proved for the unlimited BF elements without this trick, but it is so complicated in operation that we choose to avoid that way.”

now reads:

“The purpose of this transformation is to change the fractional periods into the integer periods, and to simplify the trigonometric function series.”

In the Summary and Discussion section,

“There are several errors in Ai’s article such as the integration interval, integral upper and lower limit, etc.”

now reads:

“There are some ambiguities in their article such as the integration interval, integral upper and lower limit, etc.”

In the Acknowledgements,

“This work and the authors have been supported by the following projects and grants: Natural Science Foundation of China (Project No. 11573042, 11103041, U1331113, 11427901, 11427803, U1531247); CAS President’s International Fellowship Initiative (PIFI, Basic Research Program II.16, Project No. 2017VMA0009).”

now reads:

“This work and the authors have been supported by the following projects and grants: Natural Science Foundation of China (Project No. 11573042, 11103041, U1331113, 11427901, 11427803, U1531247); CAS President’s International Fellowship Initiative (PIFI, Project No. 2017VMA0009), Basic Research Program II.16.”

In Reference 18, the page numbers “234-242” were incorrectly given as “242”.

Furthermore, References 22 and 23 were omitted and are listed below.

22. Su D. Q. A rigorous demonstration of Integrated transmission conservation of the tunable Lyot Birefringent Filter and a method to measure the integral light amounts outside the mainband. *Acta Astrophysica Sinica* **6**, 72 (1986).

23. Su D. Q. A rigorous demonstration of the conservation of Integrated transmission of the tunable Lyot Filter. *Chin. Astron. Astrophys.* **10**, 81 (1986)

As a result, the References have been renumbered.

Finally, there were a number of minor grammatical errors which have been amended.

The original Article has been corrected.

